# Temperature and time of host-seeking activity impact the efficacy of chemical control interventions targeting the West Nile virus vector, *Culex tarsalis*

**DOI:** 10.1371/journal.pntd.0012460

**Published:** 2024-08-30

**Authors:** Joshua Kalmouni, James B. Will, John Townsend, Krijn P. Paaijmans

**Affiliations:** 1 The Center for Evolution & Medicine, School of Life Sciences, Arizona State University, Tempe, Arizona, United States of America; 2 Vector Control Division, Maricopa County Environmental Services Department, Phoenix, Arizona, United States of America; 3 Simon A. Levin Mathematical, Computational and Modeling Sciences Center, Arizona State University, Tempe, Arizona, United States of America; 4 WITS Research Institute for Malaria (WRIM), Faculty of Health Sciences, University of the Witwatersrand, Johannesburg, South Africa; Kenya Agricultural and Livestock Research Organization, KENYA

## Abstract

West Nile virus (WNV) is the leading mosquito-borne disease causing-pathogen in the United States. Concerningly, there are no prophylactics or drug treatments for WNV and public health programs rely heavily on vector control efforts to lessen disease incidence. Insecticides can be effective in reducing vector numbers if implemented strategically, but can diminish in efficacy and promote insecticide resistance otherwise. Vector control programs which employ mass-fogging applications of insecticides, often conduct these methods during the late-night hours, when diel temperatures are coldest, and without a-priori knowledge on daily mosquito activity patterns. This study’s aims were to 1) quantify the effect of temperature on the toxicity of two conventional insecticides used in fogging applications (malathion and deltamethrin) to *Culex tarsalis*, an important WNV vector, and 2) quantify the time of host-seeking of *Cx*. *tarsalis* and other local mosquito species in Maricopa County, Arizona. The temperature-toxicity relationship of insecticides was assessed using the WHO tube bioassay, and adult *Cx*. *tarsalis*, collected as larvae, were exposed to three different insecticide doses at three temperature regimes (15, 25, and 35°C; 80% RH). Time of host-seeking was assessed using collection bottle rotators with encephalitis vector survey traps baited with dry ice, first at 3h intervals during a full day, followed by 1h intervals during the night-time. Malathion became less toxic at cooler temperatures at all doses, while deltamethrin was less toxic at cooler temperatures at the low dose. Regarding time of host-seeking, *Cx*. *tarsalis*, *Aedes vexans*, and *Culex quinquefasciatus* were the most abundant vectors captured. During the 3-hour interval surveillance over a full day, *Cx*. *tarsalis* were most-active during post-midnight biting (00:00–06:00), accounting for 69.0% of all *Cx*. *tarsalis*, while pre-midnight biting (18:00–24:00) accounted for 30.0% of *Cx*. *tarsalis*. During the 1-hour interval surveillance overnight, *Cx*. *tarsalis* were most-active during pre-midnight hours (18:00–24:00), accounting for 50.2% of *Cx*. *tarsalis* captures, while post-midnight biting (00:00–06:00) accounted for 49.8% of *Cx*. *tarsalis*. Our results suggest that programs employing large-scale applications of insecticidal fogging should consider temperature-toxicity relationships coupled with time of host-seeking data to maximize the efficacy of vector control interventions in reducing mosquito-borne disease burden.

## Author summary

Mosquito-borne pathogens such as West Nile virus (WNV) are expected to increase due to climate change, and public health programs rely on insecticides to reduce mosquito vector populations. Insecticidal fogging is often conducted during post-midnight hours when temperatures are coldest. Temperature has been shown to impact the toxicity of insecticides, but this effect is understudied, particularly for WNV vectors. The researchers aimed to test the effect of temperature on the toxicity of two common insecticides to *Culex tarsalis*, a primary mosquito vector of WNV, and to identify when local vectors were actively seeking a host to bite. The research location was a productive riparian (wetland) area adjacent to many urban centers in Arizona. The researchers found that both insecticides became less toxic at colder temperatures and that early evening accounted for a significant portion of captures, yet is outside of normal fogging hours. Human outdoor activities also increase during these hours, which may result in elevated risk of disease transmission. This research serves to improve our understanding and usage of insecticides to reduce disease burden. As insecticides are used globally, the findings of this research may relate to all mosquito-borne diseases, including malaria, and are relevant for agricultural pest management.

## Introduction

Vector-borne diseases remain substantially burdensome globally, accounting for over 17% of all infectious diseases and resulting in more than 700,000 deaths annually [1]. The most prevalent mosquito-borne pathogen in the United States is West Nile virus (WNV), principally vectored by *Culex tarsalis* [2,3], in addition to *Culex quinquefasciatus* and *Culex pipiens* [1,4,5]. The Pacific Southwest has a particularly high incidence of WNV, Arizona being among the states with the highest burden [6], accounting for over half of the cases reported in the United States in 2021, with 1,476 cases (~86% of all Arizona cases) originating in Maricopa County alone [7]. Arizona also contains large populations of *Aedes vexans*, a competent vector of Zika virus [8], dengue [9], and WNV [10,11], but is principally a common flood-water nuisance mosquito with sparse (current) evidence to indicate a non-negligible contribution to the transmission of these arboviruses in nature. Alarmingly, much of the Southwestern United States also has well-established populations of *Aedes aegypti*, the primary vector of many (re)emerging arboviruses such as dengue, Zika, and chikungunya [12]. Arizona lies at the forefront of the establishment of these diseases in the United States [13]. For instance, transmission of dengue regularly occurs just miles from the Arizona-Mexico border [14], and local transmission was observed in 2022 [15]. No prophylactics or drug treatments exist for these arboviruses, and disease control programs often rely on the use of insecticides. Chemically treating and reducing the availability of oviposition sites (and thus, larval habitats) relies heavily on community engagement due to the nature of the aquatic habitats of these mosquito species (i.e. many larval habitats are found on private property). For example, water-holding containers such as plastic tanks, water storage jars, flower and plant vases, rubber tires, etc. are typical larval habitats for *Aedes* species [16]. Whereas agricultural sites, wetlands, riparian zones (particularly relevant for *Culex tarsalis*, which are most abundant in rural habitats [2,17–21]), storm drains, and unmaintained or abandoned pools, known as ‘green pools’, are typical larval habitats for *Culex* species [16]. While alternative approaches are becoming more prominent (such as genetically modified mosquitoes, irradiated mosquitoes, and mosquitoes infected with *Wolbachia* [22]), insecticides are and will likely remain the primary method of disease prevention for some time–in part due to their existing availability and establishment in agricultural pest control as well as vector control globally. Barring significant insecticide-resistance, insecticides can be highly effective in reducing mosquito numbers and subsequently lower the incidence of disease due to the nonlinear effect of mosquito reductions on disease transmission [23].

In Maricopa County, AZ, insecticides are deployed through truck-mounted mosquito fogging (calibrated to control for windspeed and drift) when (i) an exceedance of mosquito abundance occurs, or (ii) a WNV positive female mosquito is detected through routine laboratory screening. Maricopa County Environmental Services Department (MCESD) utilizes over 800 surveillance traps weekly, distributed across the county [24], and deploys traps in other areas based on mosquito complaints received. This proactive approach allows MCESD to dynamically monitor mosquitoes and mosquito activity county-wide. Their surveillance system is designed such that a vector control response, typically in the form of insecticide fogging, can be implemented in the area if a trap has met a particular trigger (such as an exceedance over an abundance threshold, or an arboviral-positive sample is identified). Fogging events are conducted exclusively between the hours of 00:00 and 05:00. As a result, mosquitoes are exposed to the insecticide during the coldest portion of the diel temperature cycle. This is important as the efficacy of public health insecticides depends, to a large extent, on local temperature conditions [25–29] in addition to i) other local environmental conditions [30], such as wind [31], precipitation [32,33], and UV exposure [34,35], ii) mosquito physiological factors, such as behavioral [36], metabolic [37], altered target site [38,39], and penetration resistance [40], and iii) the application of insecticide (quality, timing, dissemination, etc.) [41,42]. Given that fogging is the core vector control approach of the MCESD, below we focus on the impact of temperature—which can vary widely during a single day in the Phoenix metropolitan area [43]—and mosquito activity patterns as to better understand the mosquito-insecticide interactions pertinent to fogging.

Regarding temperature’s influence on the efficacy of vector control, ectothermic metabolism is inferred to be an important factor in this relationship given its relevance in the modes of action for many of the major insecticide classes. Temperature’s specific effect on metabolic rate, however, is obscured. Intuitively, it is generally argued that ectothermic metabolism increases with temperature [44] under the postulation that, within thermal margins, rising temperature increases cellular activity and therefore yields increased cellular metabolism. Consequently, as metabolic rates increase, insecticide degradation may hasten and reduce exposure times which may be particularly effective in vector populations with metabolic resistance. Warmer temperatures may also induce cross-tolerance to insecticides [45]. However, this increase in metabolic rate may increase the uptake of insecticides, resulting in a higher or hastened dose. The relationship between temperature and ectothermic metabolism isn’t always clear (or linear) [46], and it has been shown that some insect populations from high-altitude and cooler sites have significantly higher metabolic rates compared to populations from warmer sites, suggesting this difference is due to the need for organisms to thermally compensate in cold climates [47–49].

In addition to climatic factors, host-seeking mosquito flight behavior, the period with the highest likelihood of substantial exposure to insecticidal fogging, remains unknown for key vector species in the Phoenix area. Observed flight (i.e., host-seeking) patterns of *Ae*. *aegypti* in other parts of the US indicate that this species is mostly active during the late afternoon-early evening [50,51]. Diel activity patterns of *Ae*. *aegypti* populations in Miami, FL, and Brownsville, TX, showed continuous activity throughout the day, but with significantly elevated peaks during the mornings and evenings [52,53]. Mutebi et al. [53] also found that *Ae*. *aegypti* diel activity significantly differed within seasons and trap locations, but not in the overall patterns within cities. Observed flight patterns of *Culex* spp. in South Carolina indicate that this genus is most-active during the first two hours following sunset and from midnight to 04:00 [51]. *Cx*. *quinquefasciatus* captured in Brownsville, TX, displayed steady activity throughout dusk to dawn [52]. However, these host-seeking patterns are contextual to geographical location (and their associated environmental conditions and factors), seasonality, and species [50,54–59], stressing the necessity for vector control programs and research partners to investigate their local conditions to tailor control methods, including educative public health communication [60], accordingly.

The aim of this study was to illustrate the potential impact of temperature—insecticide toxicity interactions as well as mosquito activity patterns on vector control efficacy in the Phoenix metropolitan area. Utilizing a temperature range that is ecologically relevant for vector mosquito species in Phoenix, and the WHO tube bioassay for insecticide resistance monitoring, we compared malathion and deltamethrin toxicities to *Cx*. *tarsalis* at three temperatures (15, 25, 35°C). Temperature can influence the efficacy of chemical control. Known as the temperature coefficient (TempCo) of insecticide toxicity, this value can be negative (i.e., increases in toxicity as temperature decreases), positive (i.e., increases in toxicity as temperature increases), but may not consistently be either value. Little progress has been made in quantifying this relationship for widely used insecticides and many disease vectors. The current research on this topic has observed different patterns, even within pyrethroids and on the same species complex [25,27], and to our knowledge, has not been done for malathion and deltamethrin and *Cx*. *tarsalis*. The direction and magnitude of the TempCo unsurprisingly differs between species and insecticide [25–29]. Naturally, as environments and climate also vary in areas of disease transmission risk, quantifying the TempCo for insecticides on local vectors should be considered essential information for vector control programs.

To our knowledge, this is the first examination of temperature’s impact on the insecticide susceptibility of *Cx*. *tarsalis* vectors in the United States, and the first collation of the timing of mass-insecticide application and mosquito flight behavior in the area.

## Methods

### Mosquito collections for the insecticide susceptibility tests

Mosquito immatures were collected at several locations within the Salt River Pima Maricopa Indian Community (SRPMIC) from May-June 2021, just prior to the start of the monsoon season. Specifically, these sites were MCESD adult surveillance trap locations which receive high capture rates annually. The SRPMIC, being adjacent to flood-irrigated farmland, riparian zones, and storm drainage systems, is simultaneously a suitable habitat for many bird species and mosquito populations. WNV-positive mosquitoes are routinely sampled from this area [61]. Additionally, it is near several urban centers, making it a high-risk area for potential arboviral disease spillover.

Twice weekly, mosquito larvae and pupae were collected from natural water bodies taken from the SRPMIC using a standard dipper (Bioquip Products Inc., CA), where its entire contents (i.e., water and debris of the dipper) were then transferred into 18 oz Whirl-Pak polyethylene bags (4 ½ x 9” White Block Whirl-Pak Bags). Upon returning to the laboratory, the contents of the Whirl-Pak bags were emptied into emergence cups (Bioquip Products Inc., CA) and naturally reared to adulthood under ambient indoor conditions. Newly emerged adults were released into experimental cages daily to guarantee the age range of the adults per experimental replicate of 2–5 days old. Mosquitoes in each cage were provided *ad libitum* access to water and 10% sucrose solution, refreshed daily. Immature collections occurred simultaneously with adult collections (see ‘Mosquito time of host-seeking’ below).

### Insecticide susceptibility tests

#### Preparation of insecticide-treated papers

The insecticide susceptibility tube bioassay of the World Health Organization (WHO) was chosen, which is an open system (i.e. mosquitoes can more readily experience the environmental temperature and humidity) in contrast with the CDC bottle bioassay method, which is fully closed.

The organophosphate malathion and the pyrethroid deltamethrin (Sigma-Aldrich, Pestanal, analytical standard, 36143-100MG and 45423-250MG, respectively) were selected to be used in this study as they are typically the active ingredient among the majority of prequalified mosquito fogging products approved for public health use [62]. The WHO presents insecticide concentrations used in their bioassay as the percentage of active ingredient per unit of volume of carrier (0.7mL) on filter paper, cut to 15x12cm (Whatman WHA1001929). For both insecticides, three concentrations (of low, intermediate, and high doses: 0.03, 0.095, 0.3% for deltamethrin; 0.8, 1.78, 8% for malathion) in addition to a control (oil only), were selected based on official WHO discriminating dose recommendations [63]. According to the WHO, the discriminating dose of deltamethrin is 0.05% for *Anopheles* spp., and 0.03% for *Aedes* spp. The discriminating dose of malathion is 5% for *Anopheles* spp., and 0.8% for *Aedes* spp. At the time of this study, official discriminating concentration information was not available for *Culex* spp., and as such, concentrations of both insecticides were selected to span (and extend) the range of concentrations for the *Anopheles* and *Aedes* genera. The WHO has since released discriminating concentrations for malathion and deltamethrin on susceptible *Cx*. *quinquefasciatus*, which are 5% and 0.025%, respectively [64]. The use of high, intermediate, and low concentrations (to WHO tube bioassay standards) was intended to investigate temperature’s effect across a gradient of concentrations. Mosquito time of host-seeking activity is expected to impact concentration exposures, and varying concentrations of insecticides from truck mounted fogging are likely to occur, given the decrease in atmospheric droplet density over relatively short distances during outdoor space spray operations [65].

#### Mosquito exposures

Using the WHO tube bioassay, about 25 2–5 day old adult female mosquitoes were acclimatized in holding tubes to one of the three temperatures (see section below) for 1hr, preceding the insecticide exposure for an additional 1hr at the same temperature. One replicate of each concentration was tested at each temperature during an experimental run, and this process was repeated 4 to 5 times (depending on the insecticide). This resulted in 12 tubes tested per run (4 concentrations of insecticides including a control, across 3 temperatures). Tubes were randomized during each run, regarding the start of the acclimatization and thus exposure period. After the 1hr exposure, the number of moribund or dead mosquitoes was scored and all mosquitoes were transferred back to the holding tube that was transferred into the post-exposure environmental chamber (malathion: 26.7°C ± 0.6 (SD), 72.7% RH ± 2.9 (SD); deltamethrin: 26.8±0.5°C, 74.1±1.8% RH. Mosquitoes had *ad libitum* access to 10% sucrose solution to assess mortality 24 hours after exposure. Survivors were killed and all mosquitoes identified to species through light microscopy by MCESD experts.

#### Temperature treatments

Insecticide susceptibility tests were conducted at three different temperatures (malathion: 16.7±0.2°C, 70.8±2.4% RH; 24.9±0.7°C, 66.0±2.3% RH; 34.4±0.8°C, 71.1±3.3% RH; deltamethrin: 16.6±0.2°C, 72.8±2.1% RH; 25.1±0.6°C, 66.3±2.7% RH; 34.6±0.7°C, 70.2±3.5% RH. Climate chambers were constructed using polystyrene housing (MateriPolar Tech 266C Thermo Chill Insulated Carton, 19” x 12” x 16”), lined with heat cable (Zoo Med Reptile Heat Cable 15 Watts, 11.5 feet) and connected to humidifiers (Coospider; Model, 15hf98-4h287). An LED light with an automatic on/off timer (MingDak Submersible LED Aquarium Light, 6W,11 Inch) maintained a 12:12 hr light:dark cycle into each chamber. Temperature and humidity were regulated by a temperature and humidity controller (Digiten DHTC-1011). Sensor cables and the humidifier hose were positioned in identical locations for each environmental chamber. As the ambient temperature of the laboratory was higher than 15°C, the low temperature treatment chamber was kept in a Caron Insect Growth Chamber (Model: 6025–1), programmed to 14°C. Temperature and humidity for all environmental chambers were monitored in one-minute intervals using Omega OM-92 temperature (NIST certified, accuracy +/- 0.3°C) and humidity (accuracy +/- 3% RH) loggers. Data were downloaded weekly.

### Mosquito time of host-seeking

#### 3h intervals during a full day

**Fig 1:**
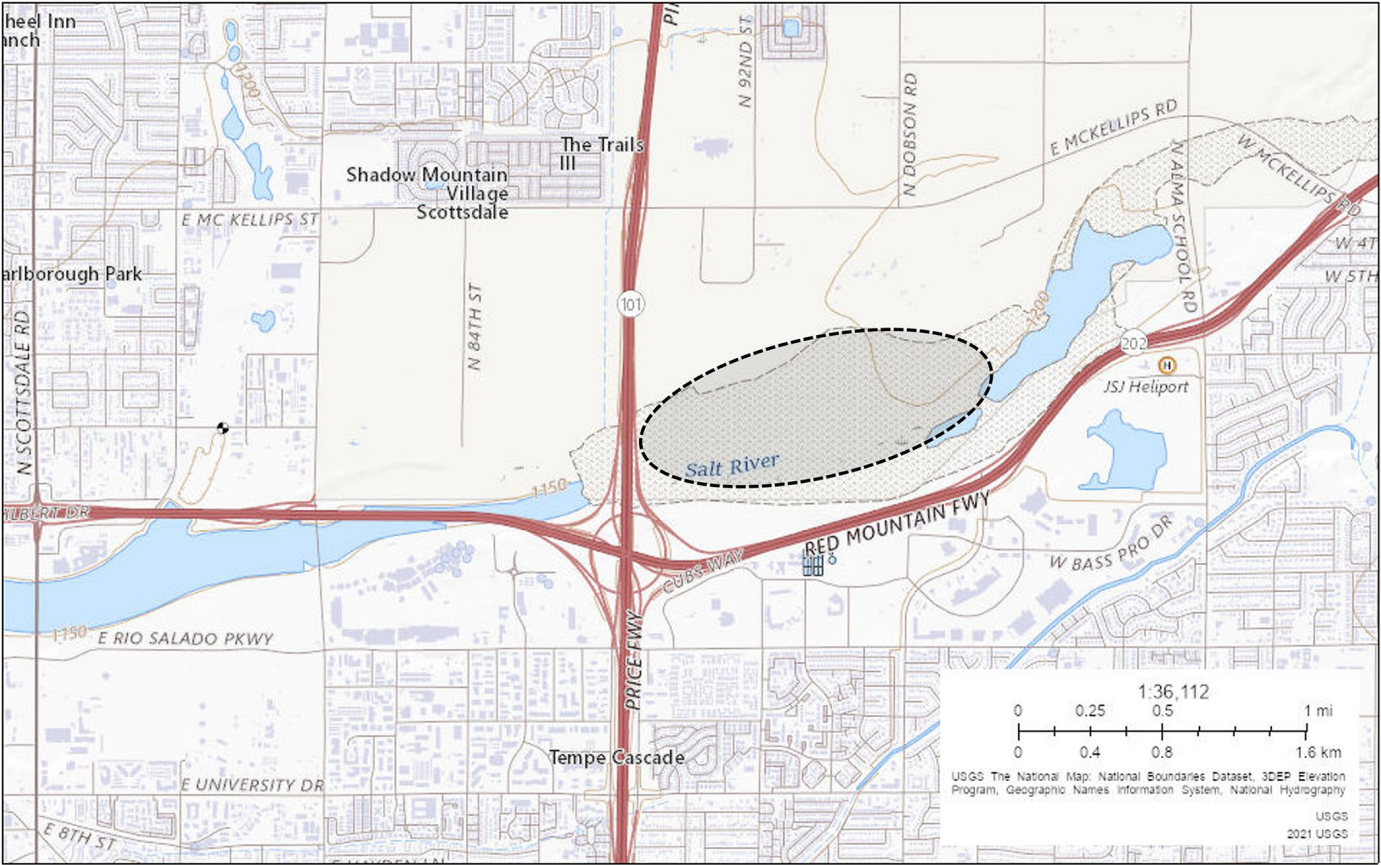
Field collection area for immature *Cx*. *tarsalis* (aim 1) and Collection Bottle Rotator traps (aim2) in the SRPMIC. Encircled area broadly denotes the coverage of the collection area used in this study. Trap locations within this area were selected based on geographical significance to Tempe, AZ, and surveillance locations already established by MCESD. Created via U.S. Geological Survey (USGS) National Map Viewer: https://apps.nationalmap.gov/viewer, accessed on 3/20/2024.

Concurrently with the immature mosquito collections for insecticide susceptibility tests, two Collection Bottle Rotators (John W. Hock Company, Gainesville, FL) were placed in different areas isolated from one another in the SRPMIC (see Fig 1) using established trap locations by the MCESD (RT242 [33.44079675, -111.8730362] and RT502 [33.44007868, -111.8889179]). The SRPMIC is known to have West Nile virus-positive vector presence [61] and substantial mosquito host-seeking activity (averaging around 1,600 captures per night between traps during the study period). Each rotator, equipped with 8 collection nets, was set to rotate every 3 hours, to evaluate mosquito activity across a full day (i.e. 24hrs). An Encephalitis Vector Survey (EVS) (John W. Hock Company, Gainesville, FL) trap was placed on top of the rotator and was baited by 3 kg of dry ice (CO_2_ sublimating via an insulated bucket with several holes in the bottom, suspended ~30 cm above the EVS trap). The dry ice was refilled (up to a maximum of 3 kg) twice daily, 10 (+/- 2) hours apart, beginning at 06:00 and again between 16:00–18:00. Each rotator was run for 3 days/week during the study period (April-May 2021), starting each day at 06:00. Total collection numbers along with species diversity and diel host-seeking activity per 3-hour period were recorded throughout the study period. Identification of captured mosquitoes was done through light microscopy by MCESD experts.

#### 1h intervals overnight

Sequentially following the 3h surveillance during the same season, four Collection Bottle Rotators were placed in different areas isolated from one another within the SRPMIC, using established trap locations by MCESD (now also including RT243 [33.44002361, -111.8861459] and RT244 [33.43954298, -111.8869971]) to assess mosquito host-seeking activity in more detail between sunset and sunrise. Rotators were designated a(n) ‘morning’ or ‘evening’ rotational schedule pseudo-randomly (i.e., an exact total of two ‘morning’ and two ‘evening’ programs were selected each night) for the duration of the study. The evening collections occurred hourly from 18:00 to 00:00; the morning collections hourly from 00:00 to 06:00. During each collection the first (i.e. net 1) and last (net 8) collection nets were not included in the analysis, as they captured mosquitoes from the time the trap was set to the start of collection, and the time between the end of the collection and the time the nets were collected, respectively. This was to remove mosquitoes which sought a blood meal at different times from the population. Each area was designated as being a(n) ‘morning’ or ‘evening’ site and subsequently alternated this designation each day for 4 days/week. Total collection numbers along with species diversity and host-seeking activity per 1-hour period were recorded throughout the study period (May-June, 2021). Identification of captured mosquitoes was done through light microscopy by MCESD experts.

### Data analysis

#### Temperature—insecticide toxicity interactions

Mortality data were analyzed using Analysis of variance (ANOVA) with a Tukey’s HSD test to assess the effect of temperature on the toxicity of insecticides to mosquitoes. Temperature was an independent variable (low, intermediate, high) and was coded as a categorical variable, with the intermediate group as the reference level. Insecticide dose was a secondary independent variable (low, intermediate, high). Experimental replicate was included as a random effect. Abbott’s formula was used to correct for natural mortality in the deltamethrin (17 and 35°C) control groups [66]. Thus, control mosquitoes were excluded from analysis, due to homogenous survival across all groups.

#### Mosquito activity patterns

Per species, trap-specific mean number of mosquitoes captured per 3-hour period were analyzed using (ANOVA) with Tukey’s post hoc. For morning and evening hourly captures (i.e. 1-hour captures), the mean site-specific counts and proportion of mean totals for species in each time-period were analyzed using ANOVA with Tukey’s post hoc.

All statistical analyses were performed in R v. 4.2.1 [67].

## Results

### The impact of temperature on the insecticide susceptibility of *Cx*. *tarsalis* to malathion and deltamethrin

**Fig 2:**
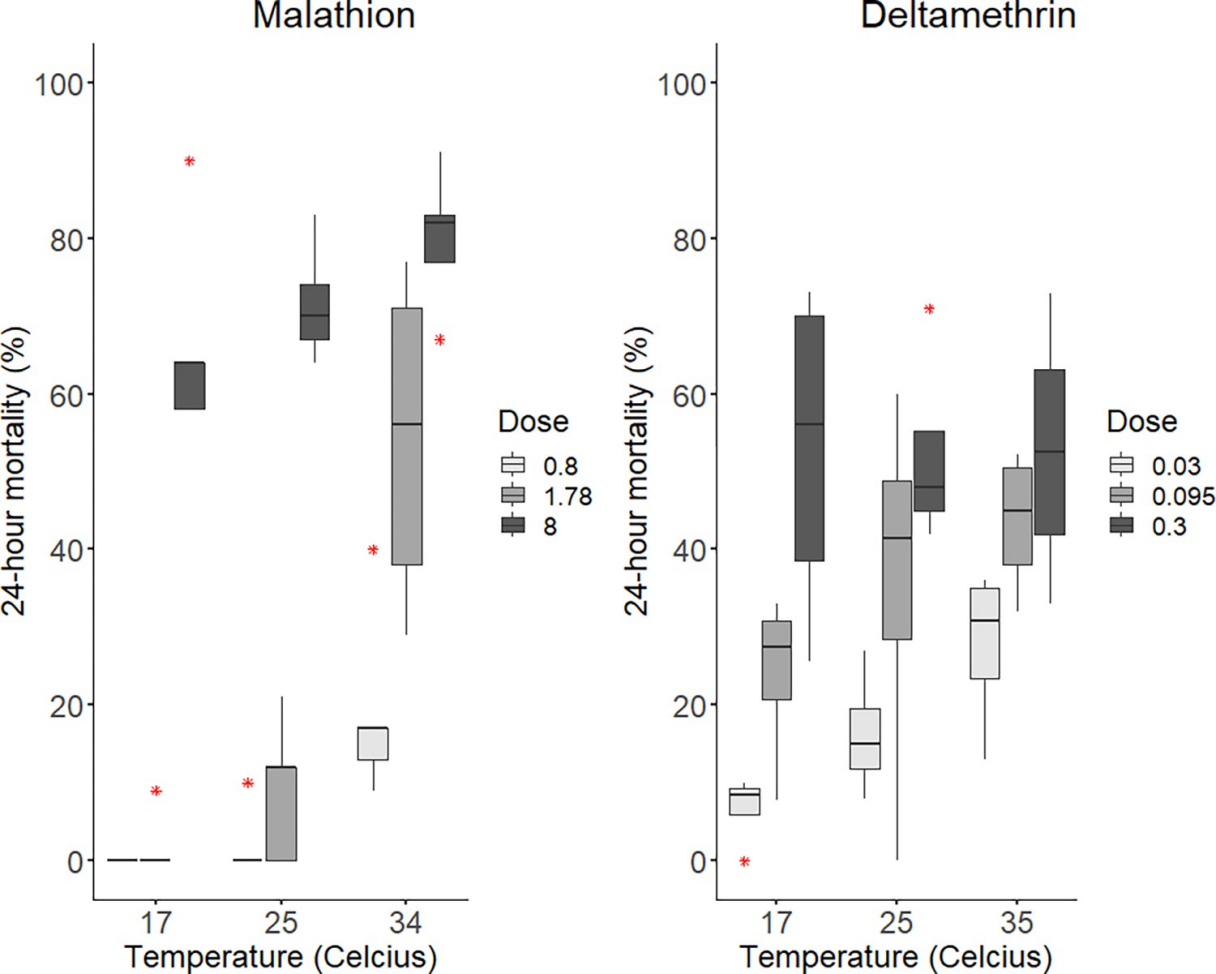
Insecticide toxicity at different temperatures to *Cx*. *tarsalis*, collected between May-June 2021. Insecticide doses are represented as the percentage of active ingredient per unit of volume of carrier (0.7mL) on the filter paper. Malathion displayed a trend of positive TempCo across doses. Similarly, deltamethrin displayed a trend of a positive TempCo at the low and intermediate dose. Abbott’s formula was used to correct for control mortality. Asterisks indicate outliers.

A total of 679 mosquitoes were tested for malathion. Temperature significantly impacted the toxicity of malathion (ANOVA: alpha = 0.05, F = 20.46, df = 2, p = 7.59e-07). ANOVA results per dose are as follows: (Fig 2; low dose ANOVA: alpha = 0.05, F = 10.05, df = 2, p = 0.00272; intermediate dose ANOVA: F = 23.14, df = 2, p = 7.62e-5; high dose ANOVA: F = 2.163, df = 2, p = 0.158). Specifically, significance was detected in the low dose between low and high temperatures (Fig 2; ANOVA: F = 9.711, df = 2, p = 0.0031, Tukey: p = 0.0045), as well as between intermediate and high temperatures (p = 0.0096). Significance was detected in the intermediate dose between low and high temperatures (p = 0.0001) and intermediate and high temperatures (p = 0.0004). At the highest dose of malathion, temperature significantly impacted the toxicity between the low and high temperatures (p = 0.0058). Mean mortality at the lowest malathion dose increased by 17% from 25 to 34°C. Mortality at intermediate malathion dose (1.78%) increased by 45% between 25 and 34°C. At the highest malathion dose (8%), mortality increased by about 15% from 17 to 34°C.

A total of 361 mosquitoes were tested for deltamethrin. Temperature significantly impacted the toxicity of deltamethrin in the low dose (Fig 2; low dose ANOVA: alpha = 0.05, F = 5.617, df = 2, p = 0.0261), but not in the intermediate (p = 0.342) or high (p = 0.996) doses. Specifically, significance was detected between the low and high temperatures at the low dose (Tukey: p = 0.0231). Mean mortality at the lowest deltamethrin dose increased by more than double at each temperature interval, showing a 45% increase in mortality at 35°C compared to 17°C. Mean mortality at the intermediate dose showed a steady increase of about 10% with increasing temperature.

### Time of host-seeking

**Fig 3:**
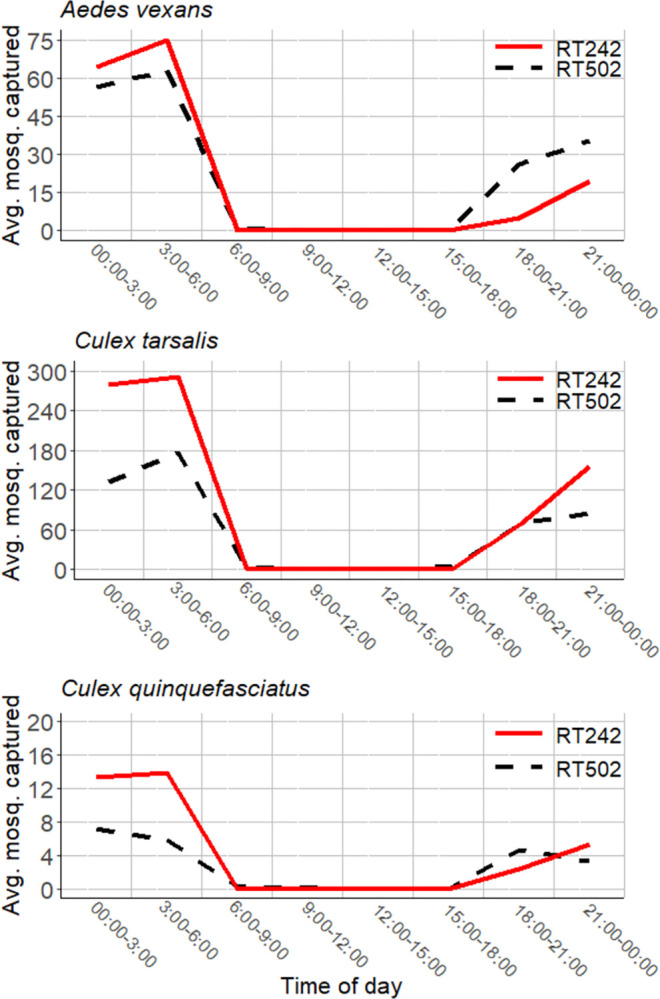
The average number of female mosquitoes caught at each site (referred to as RT242 and RT502 by MCESD, in the SRPMIC) per 3-hour period from April to May 2021. *Cx*. *tarsalis* was the most abundant species captured. Both sites reflect that the most abundant mean captures occurred between the hours of 00:00–06:00.

A total of 18 days of 24-hour surveillance at 3-hour intervals were recorded. *Cx*. *tarsalis* and *Ae*. *vexans* were the most abundant species (Fig 3). During this period, 30,137 female mosquitoes were captured, consisting of: 22,892 *Cx*. *tarsalis*, 6,225 *Ae*. *vexans*, and 1,020 *Cx*. *quinquefasciatus*. Regarding the 3-hour surveillance data, time of day (3-hour blocks) (ANOVA: alpha = 0.05, F = 24.1855, df = 7, p = < 2.2e-16) and trap location (ANOVA: alpha = 0.05, F = 8.5363, df = 1, p = 0.003766) were significant for *Cx*. *tarsalis* captures. Time of day was significant for *Cx*. *quinquefasciatus* captures (ANOVA: alpha = 0.05, F = 8.5559, df = 7, p = 1.683e-09), and time of day was significant for *Ae*. *vexans* captures (ANOVA: alpha = 0.05, F = 11.0596, df = 7, p = 2.439e-12).

During the 3-hour interval surveillance over a full day, daytime biting (06:00–18:00) accounted for 259 captures (0.86%) consisting of: 232 *Cx*. *tarsalis* (1.01% of all *Cx*. *tarsalis* captures); 18 *Ae*. *vexans* (0.29% of *Ae*. *vexans* captures); and 9 *Cx*. *quinquefasciatus* (0.88% of all *Cx*. *quinquefasciatus* captures). Pre-midnight nighttime biting (18:00–24:00) accounted for 8,696 captures (28.85%) consisting of: 6,864 *Cx*. *tarsalis* (29.98% of *Cx*. *tarsalis*); 1,547 *Ae*. *vexans* (24.85% of *Ae*. *vexans)*; and 285 *Cx*. *quinquefasciatus* (27.94% of *Cx*. *quinquefasciatus*). Post-midnight nighttime biting (00:00–06:00) accounted for 21,182 captures (70.29%) consisting of: 15,797 *Cx*. *tarsalis* (69.01% of *Cx*. *tarsalis*); 4,659 *Ae*. *vexans* (74.84% of *Ae*. *vexans)*; and 726 *Cx*. *quinquefasciatus* (71.18% of *Cx*. *quinquefasciatus)*. Peak post-midnight nighttime biting occurred between 03:00 and 06:00, accounting for 11,247 captures (37.32%) consisting of: 8,407 *Cx*. *tarsalis* (36.72% of *Cx*. *tarsalis*); 2,485 *Ae*. *vexans* (39.92% of *Ae*. *vexans*); 355 *Cx*. *quinquefasciatus* (34.80% of *Cx*. *quinquefasciatus)*.

**Fig 4:**
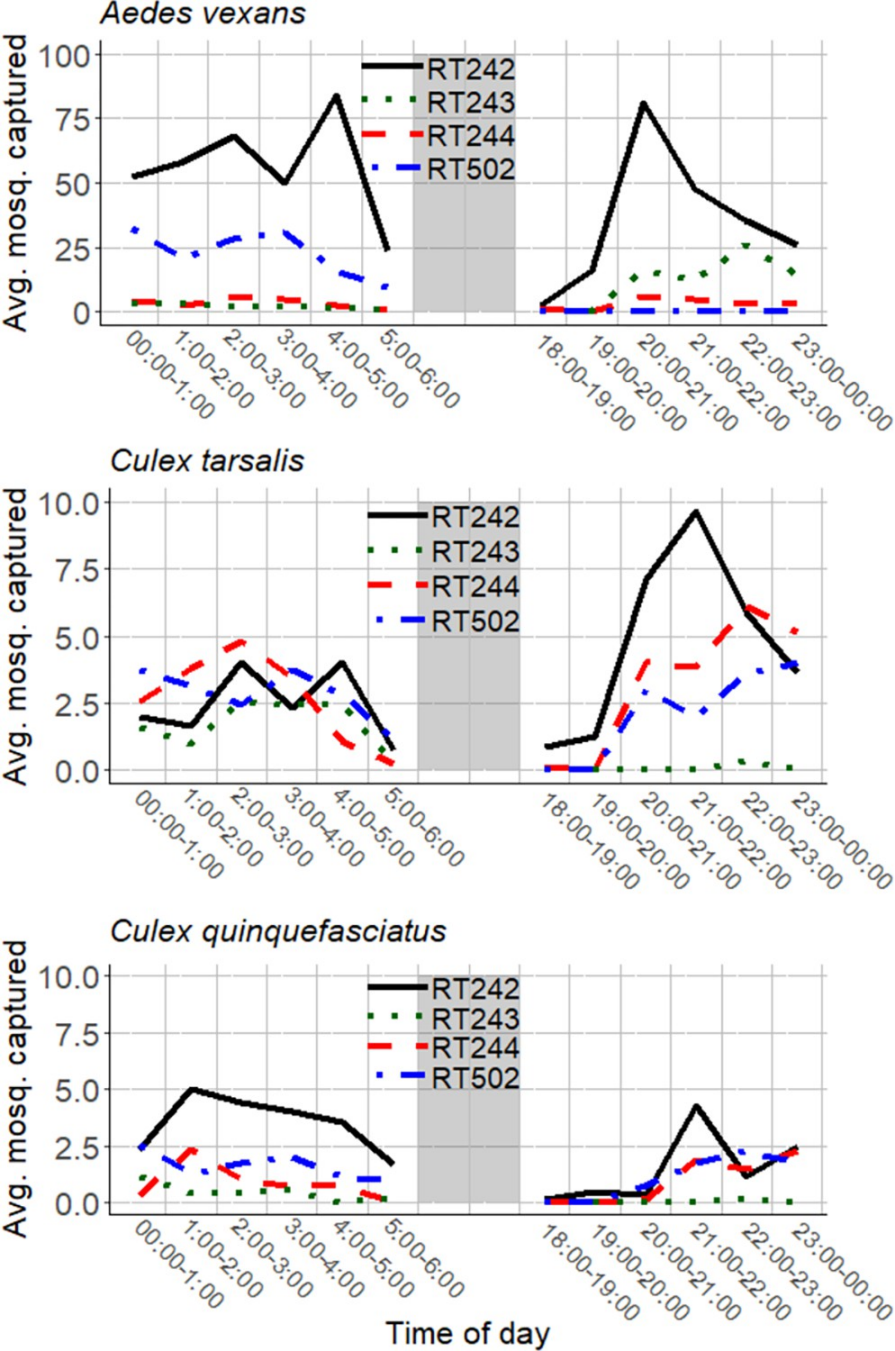
The average number of female mosquitoes caught at each site (referred to as RT242, RT243, RT244, and RT502 by MCESD, in the SRPMIC), per 1-hour period during morning (00:00–06:00) or evening (18:00–24:00) hours in the SRPMIC (May–June 2021). *Ae*. *vexans* was the most abundant species captured. Average peak times were dependent on species and trap site, indicating that evening hours are not inconsequential for host-seeking behavior.

A total of 9 nights of hourly surveillance were recorded. *Ae*. *vexans* was the most abundant species (Fig 4). During this period, 13,857 female mosquitoes were captured, consisting of: 11,569 *Ae*. *vexans*, 1,542 *Cx*. *tarsalis*, and 746 *Cx*. *quinquefasciatus*. Hourly (morning and evening) time of day was significant for *Ae*. *vexans* captures (ANOVA: alpha = 0.05, F = 2.3942, df = 11, p = 0.007147), *Cx*. *tarsalis* captures (ANOVA: alpha = 0.05, F = 2.5418, df = 11, p = 0.004221), and *Cx*. *quinquefasciatus* captures (ANOVA: alpha = 0.05, F = 2.4344, df = 11, p = 0.006198).

During this period, morning biting (00:00–06:00) accounted for 5,058 captures (63.88%) consisting of: 4,268 *Ae*. *vexans* (65.72% of *Ae*. *vexans*); 467 *Cx*. *tarsalis* (49.79% of *Cx*. *tarsalis*); and 323 *Cx*. *quinquefasciatus* (66.46% of *Cx*. *quinquefasciatus*). During the morning hours, the least amount of females across all species were captured between 05:00 and 06:00 (338), while most were captured between 02:00 and 03:00 (1,052). Evening biting (18:00–24:00) accounted for 2,860 captures (36.12%) consisting of: 2,226 *Ae*. *vexans* (28.11% of *Ae*. *vexans*); 471 *Cx*. *tarsalis* (50.21% of *Cx*. *tarsalis*); and 163 *Cx*. *quinquefasciatus* (33.54% of *Cx*. *quinquefasciatus*). During the evening hours, the least amount of females across all species were captured between 18:00 and 19:00 (35), while the most were captured between 20:00 and 21:00 (912).

## Discussion

The aim of this study was to assess the potential impact of temperature—insecticide toxicity interactions as well as mosquito activity patterns on the efficacy of insecticidal fogging activities in the Phoenix Metropolitan Area. Malathion displayed a positive TempCo (i.e. higher mortality rates at higher temperatures) on *Cx*. *tarsalis* at all doses. Temperature did not impact deltamethrin toxicity at the highest dose but did significantly impact deltamethrin toxicity at the lowest dose and displayed indicators of a positive TempCo at the intermediate dose (Fig 2). Early morning biting (00:00–06:00) accounted for 70.28% of mosquito captures during the 3-hour interval surveillance and 63.88% of captures during the hourly (morning/evening) surveillance. During the 3-hour surveillance, *Cx*. *tarsalis* were mostly active during (00:00–06:00) (69.0%), followed by (18:00–24:00) (30.0%), with the rest biting during daytime hours (1.0%). During the hourly (morning/evening) surveillance, *Cx*. *tarsalis* were mostly active during the hours of (18:00–24:00) (50.2%), followed by (00:00–06:00) (49.8%).

### Temperature-toxicity

Temperature is known to impact the toxicity of insecticides on mosquito populations [25–29]. As the concentrations used in this study exceed diagnostic concentrations for susceptible *Cx*. *quinquefasciatus* (by up to ten-fold), with mortality never reaching 100%, our data suggest that according to WHO methods for insecticide resistance monitoring, it is likely these field-collected *Cx*. *tarsalis* are resistant to these insecticides. Malathion generally displays a positive TempCo [68], as was observed in this study. As an acetylcholinesterase inhibitor, it prevents muscular neurotransmission from ceasing activation [69]. Temperature affects acetylcholinesterase activity as well as muscular neurotransmission dynamics [69,70]. The process of chemical modification (i.e., chemical changes in the insecticide compound) within the organism, called biotransformation, is reduced at colder temperatures. The compounds resulting from biotransformation of organophosphates are suggested to be more toxic than the original compound [71]. Thus, at colder temperatures, the reduction in the rate of biotransformation would subsequently yield elevated levels of the less-toxic original compound.

Deltamethrin, a type-2 pyrethroid, disrupts nerve signal activity by delaying the closure of the sodium ion channel, a process also well-documented to be influenced by temperature [72,73]. Reduced temperatures prolong the duration of steady-state resting potential and increase the stability of open-modified sodium channels, further prolonging the duration of sodium influx and susceptibility of the nervous system to the toxicity of pyrethroids. Additionally, Hardwood et al., have also proposed that at low temperatures, the reduction in biotransformation leads to an accumulation of the original compound, which is more toxic than the compound(s) created in the process of biotransformation [71]. Our results at low-intermediate doses add to the body of evidence that do not follow this trend [25,27], implicating that the temperature-toxicity relationship of pyrethroids on mosquito vectors is complex. The TempCo of pyrethroids on *Anopheles* spp. has been observed to be positive, negative, and bi-modal in cases [25,27]. It is also possible that neural sensitivity and mosquito behavior influenced by higher insecticide dosages may supersede the impact of temperature. The number of mosquitoes tested for deltamethrin in this study was limited due to the nature of field collections, thus further studies are warranted. Additionally, the overall mortality of the deltamethrin doses were low, despite the highest dose selected in this study being more than 10 times greater than the WHO’s discriminating concentration for susceptible *Cx*. *quinquefasciatus* [64]. Further, the samples collected in our study represent just a fraction of the metapopulation and are consequently limited in their translation to the field, warranting the need for additional sampling and surveillance.

### Time of host-seeking

Diel mosquito host-seeking in the SRPMIC indicate that during the pre-monsoon season, peak activity times across all three species observed occurred during the hours of 00:00–6:00. Interestingly, the majority of *Cx*. *tarsalis* captured during the 3-hour interval surveillance were captured between the hours of 00:00–06:00 (69.0%), while during the 1-hour surveillance, the slight majority of *Cx*. *tarsalis* were captured during the evening hours of 18:00–24:00 (50.2%). This deviation from the 3-hour interval observations highlights the importance to monitor diel activity year-round to better capture and quantify these patterns, as seasonality (i.e. the 1h surveillance followed the 3h surveillance in time) can impact species behaviors [51,54,55]. While 64–70% of captures are accounted for during the early morning hours, our data show that 30–36% of captures are outside of these hours–primarily occurring in the late evening hours. Of the early morning hours, the period between 05:00 and 06:00 accounted for the least number of captures, whereas most captures occurred during the period between 02:00 and 03:00, suggesting that morning-fogging would have been most optimal during this hour. Of the evening hours, the period between 18:00 and 19:00 accounted for the least number of captures, whereas most captures occurred during the period of 20:00 and 21:00 across all species, suggesting that evening-fogging would have been most optimal during this hour. Moreover, temperatures between 20:00 and 21:00 are warmer compared to 02:00 and 03:00, relevant for fogging with TempCo in mind.

Despite the potential impact of TempCo on the efficacy of employed insecticides on these vectors, the fogging window may appear to capture the period of peak host-seeking, but if residual efficacy is shortened due to any number of factors (such as increased vegetation coverage, which is often correlated with mosquito abundance [74]), the precise timing of fogging is likely critical to achieve optimal results.

Fogging treatments are effective as long as droplets remain airborne. However, droplets will fall and disperse onto objects or dissipate into the atmosphere [75]. Droplet mass dictates the rate at which they fall, and according to the WHO, this rate can vary considerably–ranging from hours to mere seconds with larger droplets falling more quickly. However, dry climates (such as Arizona) also impact the evaporative rate of the diluent used to carry the insecticide, subsequently shrinking the droplet size, and risking dissipation (for example, droplets smaller than 5 μm in diameter will be affected by the air turbulence created by a mosquito’s flight, thus limiting contact [75]). Since insecticide droplets do not remain airborne (or active) indefinitely, the timing of fogging is crucial to reduce the impact of the decay of efficacy (for example, fogging at 01:00 does not guarantee that mosquitoes active at 05:00 would be exposed to the same potency and subsequently killed, despite being within the fogging window (00:00–05:00) of MCESD’s protocol). To illustrate, for ultra-low volume (ULV) fogging studies conducted in Puerto Rico [76] and in Surinam [77], *Ae*. *aegypti* suppression may have been limited by asynchrony between the spray time and flight activity [78].

ULV fogging residual efficacy is dependent on numerous factors such as: wind speed, obstructions, vegetation, road network coverage, spray concentration, flow rate, droplet size, temperature, and timing (especially relevant for flight activity behaviors: when mosquitoes are primarily resting at the time of application, their exposure to the insecticide can be dramatically reduced) [75,79–81]. Vector control programs should consider droplet size and the environmental conditions which affect fogging dynamics (including droplets) to maximize efficacy.

In 2006, Reddy et al. studied the effects of ground applications (i.e., truck-mounted fogging) to suppress *Culex* vectors and subsequently reduce WNV transmission [79]. They found that while the vector populations were susceptible to the insecticide and that the road network was generally adequate with coverage, poor results from the application method failed to reduce WNV transmission [79]. Malathion control had decreased by up to 56% when applied in areas of vegetation (compared to open fields) [82]. Similarly, Barber et al, found that Permanone 30:30, a type-1 pyrethroid, achieved 95% mortality in the open but no better than 34% mortality in vegetated sites [83]. Accompanying the factor of timing, fogging applications can wildly vary in their success. Effectiveness of ULV spraying in vegetated habitats may be reduced by vegetation acting as a filtration of the spray, reducing the amount of insecticide available for mosquito uptake, and by reducing wind speed, similarly reducing uptake [78].

In addition, mosquito factors, such as blood feeding status, mosquito age, and metabolic rate will influence insecticide efficacy. Blood feeding and blood meal digestion reduce insecticide susceptibility [84–87], conferring varying degrees of resistance depending on species, age, insecticide, and stage of blood meal digestion [84]. Blood-fed mosquitoes may be more resistant to insecticides due to an increase in metabolic activity, leading to the increased systemic expression of detoxification enzymes [85]. Additionally, blood-fed mosquitoes may also be less sensitive to temperatures, via a protective heat shock protein response [88], which may also contribute to insecticide resistance depending on the TempCo of an insecticide. However, this interaction is not well known and should be investigated in future studies.

While one of the central aims of this study was to quantify the time of host-seeking activity in the study area, a limitation of it was the absence of simultaneous quantification of oviposition-seeking behavior, which would yield additional information of the overall flight activity of local vectors. However, our results reflect existing literature, particularly regarding general times of host-seeking behaviors of these vectors. Importantly, this also means that many vector control programs that fog only once per day/night may be missing a significant opportunity of efficacious spraying if tailoring methods toward local peak host-seeking data [52]. With approximately 30% of mosquito vectors active during the evening hours in our study, it is prudent to recognize that this is an interval of concern with regard to disease transmission risk. This is particularly troubling when considering that human outdoor activity generally increases during evening hours, and there is little human activity between midnight and 06:00 [89]. WNV (and other arboviral) spillover risk will likely be disproportionately elevated during these hours. Having said that, this study was limited spatially (albeit laboratory-confirmed WNV infected mosquitoes frequently inhabit the area [61]) and to the pre-monsoon season, just before the intense Arizona summer, where mosquito abundance (particularly *Cx*. *tarsalis*) is reduced [90]. Year-round surveillance, thus including the post-monsoon season when mosquito abundance increases again, is warranted.

Whilst fogging may reduce mosquito populations, it may very well not reduce disease risk if biting/activity time is genetically determined and may worsen if fogging practices have an effect of selection on biting times. Heritable biting behaviors can be subjected to selection from intervention methods, as seen in *Anopheles arabiensis* [91]. Circadian rhythms (clocks) of insects provide synchronization of key life history traits, controlling physiology and behaviors, such as host-seeking and resting patterns. The circadian clock may also influence the chronotoxicity of insecticides. For example, a link between pyrethroid-resistance and the circadian clock in *Ae*. *aegypti* has been observed by Yang et al. [92], suggesting that circadian expressions of genes are likely to be involved in insecticide detoxification processes. Disruptions of circadian clocks in *Ae*. *aegypti* were also linked to altered host-seeking behavior [93]. Variability in circadian rhythms was observed in the *Culex pipiens* complex, suggesting that genetic differences may yield differing activity patterns, independently of seasonality [94]. The complete role of circadian rhythms and their relation to vector control efforts is not fully understood and requires future investigation. Behavioral resistance (evolution of behavioral traits in response to intervention selection) or resilience (biting behavior plasticity) are also dynamic factors of interventions that may affect fogging efficacy. Again, year-round sampling (or at minimum, the full mosquito season), will provide more robust vector-associated details. Expanding surveillance to include considerations based on epidemiological indicators (such as relevant socio-economic, land-use (urbanization), local genetics and urban pollutants (e.g., artificial light) which are likely to influence biting patterns [95]), and vegetation gradients (as vegetation cover impacts fogging efficacy [78]), in addition to refining reservoir surveillance (e.g., bird reservoirs for WNV), will improve intervention response.

### Influence of temperature on vector control

Aridification is expanding, including in the Pacific Southwest [96]. Even under moderate climate change scenarios, vector borne disease dynamics are expected to be impacted dramatically [96]. Climate change will affect the shifting of vector and disease distribution—posing logistical challenges in adequate public health response, particularly where none may have been needed before [97]. Whether climate change will impact mosquito abundance and species diversity positive or negatively is beyond the scope of this work, but warming is likely to impact the toxicity of any insecticide due to their TempCo. Temperature stress may aggravate the negative effect of insecticide on mosquitoes either by increasing their sensitivity to insecticide or enhancing insecticide toxicity. Alternatively, warmer temperature conditions may induce cross- tolerance to insecticides [98] or lead to more rapid insecticide degradation that may be beneficial to mosquitoes given shorter exposure periods [99]. It is largely unknown which effect(s) are more significant. Additional toxicokinetic processes are also impacted by temperature, and warmer temperatures can accelerate the physiological mechanisms underlying these processes. Higher temperatures can also aggravate and augment mosquito activity, increasing metabolic activity yielding elevated oxygen demand and respiration rate [100]. This may result in a greater uptake of the insecticide. As such, more research is needed to identify the intricacies driving the effect of temperature on insecticide toxicity.

Mosquitoes reared at warmer temperatures tend to progress through immature stages more quickly, resulting in smaller adults due to a reduced opportunity to accumulate mass during larval development. This can impact behavior [101] as well as insecticide susceptibility since weight is an indicator for susceptibility [102]. Broadly, regardless of size, warmer temperatures affect flight behavior (i.e., reduction in total travelable distance as well as shorter flights [101]), and mosquitoes may adapt to be active later during the night to avoid (the most) unfavorable temperatures. In the context of this study, mosquitoes are exposed to insecticide sprays during the coldest part of the day/night cycle by MCESD. This may translate into reduced toxicity in field conditions if the TempCo of the insecticide is positive. However, field studies conducted locally will ultimately show the practicality and feasibility of using chemical control with TempCo taken into consideration. As such, also understanding how climate influences the physiology and behaviors of relevant mosquito vectors could produce more optimal use of chemical control.

### Modeling and future directions

Wilke et al. have generated a model evaluating insecticidal fogging efficacy based on vector abundance capture data in Texas and Florida populations [52]. The model’s results unsurprisingly showed that fogging during peak activity time windows for relevant vector species yielded increased efficacy, which was further improved when fogging two times per day for species with bi-modal peaks or a steady abundance. This model illustrates a promising precedent toward a more efficacious use of insecticidal fogging, as with the rise of mosquito-borne disease burden and insecticide resistance globally, it is paramount to maximize the few public health insecticidal classes allowed for fogging [62,103]. Moreover, actualized repeated applications of fogging appears to be effective in increasing control [104–106]. Repeated applications could be especially useful during arboviral outbreaks or seasons known to have higher incidence of disease transmission. A further application of this model could be to couple with context-specific (i.e., local insecticides and species) TempCo data to improve model predictions in efficacy. Repeated applications should also be tailored with the incubation period of an arbovirus in mind, to avoid repeated sprays too early or too late [105].

## Conclusion

Temperature had a significant effect on the toxicity of malathion at all doses to local *Cx*. *tarsalis*, with higher mortality rates at higher temperatures. Deltamethrin was more toxic at the highest temperature in the lowest dose and displayed indicators of a positive TempCo at the intermediate dose. Concerningly, local populations of *Cx*. *tarsalis* appear to be resistant to malathion and deltamethrin, two different insecticide classes with differing target sites [64]. This sets an alarming precedent for areas which utilize insecticidal fogging as the primary method of vector control. A significant portion of mean of mosquito captures occurred during the evening hours, which may indicate higher risk of disease transmission, as human outdoor activity is also increased during these hours compared to activity between midnight and 06:00. Vector control programs could bolster current fogging operations by considering TempCo and peak time(s) of biting. If daytime fogging is an option (which may not be possible due to public sentiment [78]), insecticides that have a positive TempCo on local vectors during the warmer hours of the diel temperature cycle could be more effective, while fogging with insecticides that have a negative TempCo may be more effective during the early morning hours. Vector control programs could also consider additional strategies to address the peak overlap of human-mosquito activity, such as fogging during the daytime and before midnight to reduce disease transmission risk. Field trials of these strategies across different seasons should be conducted however, as these assumptions are based on laboratory testing of insecticide applications and other field studies that may differ from local field conditions. Lastly, utilizing locally-relevant TempCo data, as well as data on human and mosquito activity patterns [107–109] into existing models [52,110] can help improve the impact of local vector control efforts.

## Supporting information

S1 Data Insecticide data. (XLSX)

S2 Data 3h interval trap data. (XLSX)

S3 Data 1h interval trap data. (XLSX)

## References

[1] World Health Organization. Vector-borne diseases. 2020. Available from: https://www.who.int/news-room/fact-sheets/detail/vector-borne-diseases

[2] Dunphy BM, Kovach KB, Gehrke EJ, Field EN, Rowley WA, Bartholomay LC, et al. Long-term surveillance defines spatial and temporal patterns implicating *Culex tarsalis* as the primary vector of West Nile virus. Sci Rep. 2019;9:6637. doi: 10.1038/s41598-019-43246-y 31036953 PMC6488619

[3] Colpitts TM, Conway MJ, Montgomery RR, Fikrig E. West Nile virus: Biology, transmission, and human infection. Clin Microbiol Rev. 2012;25: 635–648. doi: 10.1128/CMR.00045-12 23034323 PMC3485754

[4] Vector Disease Control International. West Nile virus. 2021 [cited 21 Dec 2023]. Available from: https://www.vdci.net/vector-borne-diseases/west-nile-virus-education-and-mosquito-management-to-protect-public-health/

[5] World Health Organization. West Nile virus. 2017. Available from: https://www.who.int/news-room/fact-sheets/detail/west-nile-virus#:~:text=Mosquitoes

[6] Ruberto I, Kretschmer M, Zabel K, Sunenshine R, Smith K, Townsend J, et al. Notes from the field: An outbreak of West Nile virus—Arizona, 2019. MMWR Morb Mortal Wkly Rep. 2021;70: 123–124. doi: 10.15585/mmwr.mm7004a4 33507888 PMC7842816

[7] CDC. West Nile virus disease cases by state 2021 West Nile virus CDC. 2022 [cited 18 Oct 2022]. Available from: https://www.cdc.gov/westnile/statsmaps/preliminarymapsdata2021/disease-cases-state-2021.html#print

[8] Gendernalik A, Weger-Lucarelli J, Garcia Luna SM, Fauver JR, Rückert C, Murrieta RA, et al. American *Aedes vexans* mosquitoes are competent vectors of Zika virus. Am J Trop Med Hyg. 2017;96: 1338–1340. doi: 10.4269/ajtmh.16-0963 28719283 PMC5462567

[9] Li Y, An Q, Sun Z, Gao X, Wang H. Distribution areas and monthly dynamic distribution changes of three *Aedes* species in China: *Aedes aegypti*, *Aedes albopictus* and *Aedes vexans*. Parasit Vectors. 2023;16:297. doi: 10.1186/s13071-023-05924-9 37633953 PMC10463299

[10] Anderson JF, Main AJ, Ferrandino FJ. Horizontal and vertical transmission of West Nile virus by *Aedes vexans* (diptera: culicidae). J Med Entomol. 2020;57: 1614–1618. doi: 10.1093/jme/tjaa049 32188992

[11] Wöhnke E, Vasic A, Raileanu C, Holicki CM, Tews BA, Silaghi C. Comparison of vector competence of *Aedes vexans* Green River and *Culex pipiens* biotype pipiens for West Nile virus lineages 1 and 2. Zoonoses Public Health. 2020;67: 416–424. doi: 10.1111/zph.12700 32162489

[12] Velayudhan Raman, Yadav A, Mnzava M, Quinones Knox. Vector control operations framework for Zika virus. In: World Health Organization [Internet]. 2016 [cited 21 Dec 2023] pp. 4–10. Available from: https://www.who.int/publications/i/item/WHO-ZIKV-VC-16.4

[13] Ananth S, Shrestha N, Treviño C JA, Nguyen US, Haque U, Angulo-Molina A, et al. Clinical symptoms of arboviruses in Mexico. Pathogens. 2020;9:964. doi: 10.3390/pathogens9110964 33228120 PMC7699393

[14] Leta S, Beyene TJ, De Clercq EM, Amenu K, Kraemer MUG, Revie CW. Global risk mapping for major diseases transmitted by *Aedes aegypti* and *Aedes albopictus*. International Journal of Infectious Diseases. 2018;67: 25–35. doi: 10.1016/j.ijid.2017.11.026 29196275 PMC5976855

[15] Arizona Health Department. Public health conducting Dengue surveillance in one neighborhood. 4 Nov 2022 [cited 21 Dec 2023]. Available from: https://www.maricopa.gov/CivicAlerts.aspx?AID=2618

[16] Centers for Disease Control and Prevention. Life cycle of *Aedes aegypti* and *Ae*. *albopictus* mosquitoes. 2022. Available from: https://www.cdc.gov/mosquitoes/about/life-cycles/aedes.html

[17] DeGroote JP, Sugumaran R, Brend SM, Tucker BJ, Bartholomay LC. Landscape, demographic, entomological, and climatic associations with human disease incidence of West Nile virus in the state of Iowa, USA. Int J Health Geogr. 2008;7:19. doi: 10.1186/1476-072X-7-19 18452604 PMC2396613

[18] Larson SR, Degroote JP, Bartholomay LC, Sugumaran R. Ecological niche modeling of potential West Nile virus vector mosquito species in Iowa. J Insect Sci. 2010,10:110. doi: 10.1673/031.010.11001 20874412 PMC3016929

[19] Pitzer JB, Byford RL, Vuong HB, Steiner RL, Creamer RJ, Caccamise DF. Potential vectors of West Nile virus in a semiarid environment: Doñ a Ana County, New Mexico. J Med Entomol. 2009;46(6):1474–1482 doi: 10.1603/033.046.0634 19960700

[20] Dimenna MA, Bueno R, Parmenter RR, Norris DE, Sheyka JM, Molina JL, et al. Emergence of West Nile virus in mosquito (Diptera: Culicidae) communities of the New Mexico Rio Grande Valley. J Med Entomol. 2006; 43:594–599. doi: 10.1603/0022-2585(2006)43[594:EOWNVI]2.0.CO;2 16739421 PMC4152309

[21] Reisen WK, Lothrop HD, Wheeler SS, Kennsington M, Gutierrez A, Fang Y, et al. Persistent West Nile virus transmission and the apparent displacement St. Louis Encephalitis virus in southeastern California, 2003−2006. J Med Entomol. 2008; 45:494–508, doi: 10.1603/0022-2585(2008)45[494:pwnvta]2.0.co;2 18533445 PMC2435167

[22] Centers for Disease Control and Prevention. Emerging methods for mosquito control mosquitoes. 2022 [cited 21 Dec 2023]. Available from: https://www.cdc.gov/mosquitoes/mosquito-control/community/emerging-methods/index.html

[23] Smith DL, Battle KE, Hay SI, Barker CM, Scott TW, McKenzie FE. Ross, Macdonald, and a theory for the dynamics and control of mosquito-transmitted pathogens. PLoS Pathog. 2012; 8(4): e1002588. doi: 10.1371/journal.ppat.1002588 22496640 PMC3320609

[24] United States Census Bureau. Arizona; Maricopa County, Arizona. 2020 [cited 21 Dec 2023] pp. 7–9. Available from: https://www.census.gov/quickfacts/fact/table/maricopacountyarizona/PST045222

[25] Glunt KD, Oliver S V., Hunt RH, Paaijmans KP. The impact of temperature on insecticide toxicity against the malaria vectors *Anopheles arabiensis* and *Anopheles funestus*. Malar J. 2018;17131. doi: 10.1186/s12936-018-2250-4 29606123 PMC5879579

[26] Whiten SR, Peterson RKD. The influence of ambient temperature on the susceptibility of *Aedes aegypti* (Diptera: Culicidae) to the pyrethroid insecticide permethrin. J Med Entomol. 2016;53: 139–143. doi: 10.1093/jme/tjv159 26477050

[27] Hodjati MH, Curtis CF. Effects of permethrin at different temperatures on pyrethroid-resistant and susceptible strains of *Anopheles*. Med Vet Entomol. 1999;13: 415–422. doi: 10.1046/j.1365-2915.1999.00198.x 10608231

[28] Muturi EJ. Larval rearing temperature influences the effect of malathion on *Aedes aegypti* life history traits and immune responses. Chemosphere. 2013;92: 1111–1116. doi: 10.1016/j.chemosphere.2013.01.055 23419321

[29] Muturi EJ, Lampman R, Costanzo K, Alto BW. Effect of temperature and insecticide stress on life-history traits of *Culex restuans* and *Aedes albopictus* (Diptera: Culicidae). J Med Entomol. 2011;48: 243–250. doi: 10.1603/ME10017 21485359

[30] Rhodes LA, McCarl BA. An analysis of climate impacts on herbicide, insecticide, and fungicide expenditures. Agronomy. 2020;10:745. doi: 10.3390/agronomy10050745

[31] Hoffmann WC, Fritz BK, Farooq M, Cooperband MF. Effects of wind speed on aerosol spray penetration in adult mosquito bioassay cages. J Am Mosq Control Assoc. 2008;24: 419–426. doi: 10.2987/5707.1 18939696

[32] Gautam BK, Little BA, Taylor MD, Jacobs JL, Lovett WE, Holland RM, et al. Effect of simulated rainfall on the effectiveness of insecticides against spotted wing drosophila in blueberries. Crop Protection. 2016;81: 122–128. doi: 10.1016/j.cropro.2015.12.017

[33] Ivlashaya N. Effect of simulated rain on efficacy of insecticide deposits on tobacco. Crop Protection. 1993;12: 55–58. 10.1016/0261-2194(93)90020-J.

[34] Sibanda MM, Focke WW, Labuschagne FJWJ, Moyo L, Nhlapo NS, Maity A, et al. Degradation of insecticides used for indoor spraying in malaria control and possible solutions. Malar J. 2011;10:307. doi: 10.1186/1475-2875-10-307 22008292 PMC3213200

[35] Xi N, Li Y, Chen J, Yang Y, Duan J, Xia X. Elevated temperatures decrease the photodegradation rate of pyrethroid insecticides on spinach leaves: Implications for the effect of climate warming. Environ Sci Technol. 2021;55: 1167–1177. doi: 10.1021/acs.est.0c06959 33356194

[36] Gatton ML, Chitnis N, Churcher T, Donnelly MJ, Ghani AC, Godfray HCJ, et al. The importance of mosquito behavioural adaptations to malaria control in Africa. Evolution (N Y). 2013;67: 1218–1230. doi: 10.1111/evo.12063 23550770 PMC3655544

[37] Schluep SM, Buckner EA. Metabolic resistance in permethrin-resistant Florida Aedes aegypti (Diptera: Culicidae). Insects. 2021;12:866. doi: 10.3390/insects12100866 34680634 PMC8540271

[38] Dafalla O, Alsheikh A, Mohammed W, Shrwani K, Alsheikh F, Hobani Y, et al. Knockdown resistance mutations contributing to pyrethroid resistance in *Aedes aegypti* population, Saudi Arabia. Eastern Mediterranean Health Journal. 2019;25: 905–913. doi: 10.26719/emhj.19.081 32003449

[39] Rahman RU, Souza B, Uddin I, Carrara L, Brito LP, Costa MM, et al. Insecticide resistance and underlying targets-site and metabolic mechanisms in *Aedes aegypti* and *Aedes albopictus* from Lahore, Pakistan. Sci Rep. 2021;11:4555. doi: 10.1038/s41598-021-83465-w 33633183 PMC7907206

[40] Buhler W. Insecticide resistance mechanisms. [cited 21 Dec 2023]. Available from: https://pesticidestewardship.org/resistance/insecticide-resistance/insecticide-resistance-mechanisms/

[41] Jinjing Xiao, Li Chen, Fan Pan, Yajing Deng, Chenchun Ding, Min Liao, et al. Application method affects pesticide efficiency and effectiveness in wheat fields. Pest Manag Sci. 2020;76: 1256–1264. doi: 10.1002/ps.5635 31595654

[42] Tudi M, Ruan HD, Wang L, Lyu J, Sadler R, Connell D, et al. Agriculture development, pesticide application and its impact on the environment. Int J Environ ResPublic Health. 2021. 18:1112. doi: 10.3390/ijerph18031112 33513796 PMC7908628

[43] Yap TF, Decker CJ, Preston DJ. Effect of daily temperature fluctuations on virus lifetime. SciTotal Environ. 2021;789: 148004. doi: 10.1016/j.scitotenv.2021.148004 34323833 PMC8570935

[44] Riemer K, Anderson-Teixeira KJ, Smith FA, Harris DJ, Ernest SKM. Body size shifts influence effects of increasing temperatures on ectotherm metabolism. Global Ecology and Biogeography. 2018;27: 958–967. doi: 10.1111/geb.12757

[45] Meng S, Delnat V, Stoks R. Mosquito larvae that survive a heat spike are less sensitive to subsequent exposure to the pesticide chlorpyrifos. Environmental Pollution. 2020;265:114824. doi: 10.1016/j.envpol.2020.114824 32454381

[46] Clarke A, Fraser KPP. Why does metabolism scale with temperature? Funct Ecol. 2004;18: 243–251. doi: 10.1111/j.0269-8463.2004.00841.x

[47] Vorhees AS, Gray EM, Bradley TJ. Thermal resistance and performance correlate with climate in populations of a widespread mosquito. Physiol Biochem Zool. 2013;86: 73–81. doi: 10.1086/668851 23303322

[48] Lahondère C, Bonizzoni M. Thermal biology of invasive *Aedes* mosquitoes in the context of climate change. Curr Opin Insect Sci. 2022; 51: 100920. doi: 10.1016/j.cois.2022.100920 35421621

[49] Williams CM, Szejner-Sigal A, Morgan TJ, Edison AS, Allison DB, Hahn DA. Adaptation to low temperature exposure increases metabolic rates independently of growth rates. Integr Comp Biol 2016; 56:62–72. doi: 10.1093/icb/icw009 27103615 PMC4930064

[50] Smith M, Dixon D, Bibbs C, Autry D, Xue R De. Diel patterns of *Aedes aegypti* (Diptera: Culicidae) after resurgence in St. Augustine, Florida as collected by a mechanical rotator trap. JVector Ecol. 2018;43: 201–204. doi: 10.1111/jvec.12302 29757509

[51] Breidenbaugh MS, Clark JW, Brodeur RM, de Szalay FA. Seasonal and diel patterns of biting midges (Ceratopogonidae) and mosquitoes (Culicidae) on the Parris Island Marine Corps Recruit Depot. J Vector Ecol. 2009;34: 129–140. doi: 10.1111/j.1948-7134.2009.00016.x 20836813

[52] Wilke ABB, Mhlanga A, Kummer AG, Vasquez C, Moreno M, Petrie WD, et al. Diel activity patterns of vector mosquito species in the urban environment: Implications for vector control strategies. PLoS Negl Trop Dis. 2023;17: e0011074. doi: 10.1371/journal.pntd.0011074 36701264 PMC9879453

[53] Mutebi JP, Wilke ABB, Ostrum E, Vasquez C, Cardenas G, Carvajal A, et al. Diel activity patterns of two distinct populations of *Aedes aegypti* in Miami, FL and Brownsville, TX. Sci Rep. 2022;12:5315. doi: 10.1038/s41598-022-06586-w 35351905 PMC8964714

[54] Chadee DD, Tikasingh ES, Ganesh R. Seasonality, biting cycle and parity of the yellow fever vector mosquito *Haemagogus janthinomys* in Trinidad. Med Vet Entomol. 1992;6: 143–148. doi: 10.1111/j.1365-2915.1992.tb00592.x 1358266

[55] Ryan SJ, Lippi CA, Boersch-Supan PH, Heydari N, Silva M, Adrian J, et al. Quantifying seasonal and diel variation in anopheline and *Culex* human biting rates in Southern Ecuador. Malar J. 2017;16:479. doi: 10.1186/s12936-017-2121-4 29166907 PMC5700746

[56] Captain-Esoah M, Baidoo PK, Frempong KK, Adabie-Gomez D, Chabi J, Obuobi D, et al. Biting behavior and molecular identification of *Aedes aegypti* (Diptera: Culicidae) subspecies in some selected recent yellow fever outbreak communities in Northern Ghana. J Med Entomol. 2020;57: 1239–1245. doi: 10.1093/jme/tjaa024 32112094

[57] Mohammed A, Chadee DD. Effects of different temperature regimens on the development of *Aedes aegypti* (L.) (Diptera: Culicidae) mosquitoes. Acta Trop. 2011; 119:38–43. doi: 10.1016/j.actatropica.2011.04.004 21549680

[58] Chadee DD, Tikasingh ES. Diel biting activity of *Culex* (Melanoconion) *caudelli* in Trinidad, West Indies. Med Vet Entomol. 1989;3: 231–237. 2519669 10.1111/j.1365-2915.1989.tb00221.x

[59] Thavara U, Tawatsin A, Chansang C, Kong-ngamsuk W, Paosriwong S, Boon-Long J, et al. Larval occurrence, oviposition behavior and biting activity of potential mosquito vectors of dengue on Samui Island, Thailand. J Vector Ecol. 2001;26: 172–80. 11813654

[60] Potter A, Jardine A, Morrissey A, Lindsay MDA. Evaluation of a health communication campaign to improve mosquito awareness and prevention practices in western Australia. Front Public Health. 2019;7:54. doi: 10.3389/fpubh.2019.00054 30941341 PMC6433780

[61] Hepp CM, Cocking JH, Valentine M, Young SJ, Damian D, Samuels-Crow KE, et al. Phylogenetic analysis of West Nile virus in Maricopa County, Arizona: Evidence for dynamic behavior of strains in two major lineages in the American Southwest. PLoS One. 2018;13:e0205801. doi: 10.1371/journal.pone.0205801 30475820 PMC6261030

[62] World Health Organization. Vector control product list. [cited 21 Dec 2023]. Available from: https://extranet.who.int/prequal/vector-control-products/prequalified-product-list

[63] The World Health Organization. Test procedures for insecticide resistance monitoring in malaria vector mosquitoes. Second edition. 2016. Available from: https://fctc.who.int/publications/i/item/9789241511575

[64] World Health Organization. Standard operating procedure for testing insecticide susceptibility of adult mosquitoes in WHO tube tests. 2022. Available from: http://www.inreskit.usm.my

[65] Britch SC, Linthicum KJ, Wynn WW, Walker TW, Farooq M, Smith VL, et al. Evaluation of ULV and thermal fog mosquito control applications in temperate and desert environments. J Am Mosq Control Assoc. 2010;26: 183–197. doi: 10.2987/09-5948.1 20649128

[66] Abbott WS. A method of computing the effectiveness of an insecticide. J Econ Entomol. 1925;18: 265–267. 10.1093/jee/18.2.265a

[67] The R Foundation. The R Project for Statistical Computing. 2023 [cited 21 Dec 2023]. Available from: https://www.r-project.org/

[68] Dietrich JP, Van Gaest AL, Strickland SA, Arkoosh MR. The impact of temperature stress and pesticide exposure on mortality and disease susceptibility of endangered Pacific salmon. Chemosphere. 2014;108: 353–359. doi: 10.1016/j.chemosphere.2014.01.079 24559935

[69] Trang A, Khandhar PB. Physiology, acetylcholinesterase. StatPearls; 2023. 30969557

[70] Pfeifer S, Schiedek D, Dippner JW. Effect of temperature and salinity on acetylcholinesterase activity, a common pollution biomarker, in *Mytilus* sp. from the south-western Baltic Sea. J Exp Mar Biol Ecol. 2005;320: 93–103. doi: 10.1016/j.jembe.2004.12.020

[71] Harwood AD, You J, Lydy MJ. Temperature as a toxicity identification evaluation tool for pyrethroid insecticides: Toxicokinetic confirmation. Environ Toxicol Chem. 2009;28: 1051–1058. doi: 10.1897/08-291.1 19071969

[72] Buzatu S. The temperature-induced changes in membrane potential. Riv Biol. 2009;102: 199–217. 20077389

[73] Ruff RL. Effects of temperature on slow and fast inactivation of rat skeletal muscle Na channels. Am J Phys. 1999;277: 937–947. doi: 10.1152/ajpcell.1999.277.5.C937 10564086

[74] Ferraguti M, Martínez-De La Puente J, Roiz D, Ruiz S, Soriguer R, Figuerola J. Effects of landscape anthropization on mosquito community composition and abundance. Sci Rep. 2016;6:29002. doi: 10.1038/srep29002 27373794 PMC4931447

[75] World Health Organization. Space spray application of insecticides for vector and public health pest control. A practitioner’s guide. 2003. Available from: https://www.who.int/publications/i/item/who-cds-whopes-gcdpp-2003.5

[76] Fox I SP. Evaluating ultra-low volume ground applications of malathion against *Aedes aegypti* using landing counts in Puerto Rico, 1980–84. J Am Mosq Control Assoc. 1988;4: 163–7. 3193113

[77] Cator LJ, Johnson LR, Mordecai EA, El Moustaid F, Smallwood TRC, LaDeau SL, et al. The role of vector trait variation in vector-borne disease dynamics. Front Ecol Evol. 2020;8:189. doi: 10.3389/fevo.2020.00189 32775339 PMC7409824

[78] Bonds JAS. Ultra-low-volume space sprays in mosquito control: A critical review. Med Vet Entomol. 2012. 26:121–130. doi: 10.1111/j.1365-2915.2011.00992.x 22235908

[79] Reddy MR, Spielman A, Lepore TJ, Henley D, Kiszewski AE, Reiter P. Efficacy of resmethrin aerosols applied from the road for suppressing *Culex* vectors of West Nile virus. Vector-borne and Zoonotic Dis. 2006;6: 117–127. doi: 10.1089/vbz.2006.6.117 16796509

[80] Perich MJ, Davila G, Turner A, Garcia A, Nelson AM. Behavior of resting *Aedes aegypti* (Culicidae: Diptera) and its relation to ultra-low volume adulticide efficacy in Panama City, Panama. J Med Entomol. 2000; 37:541–546 10916294 10.1603/0022-2585-37.4.541

[81] Andis MD, Sackett SR, Carroll MK, Bordes ES. Strategies for the emergency control of arboviral epidemics in New Orleans. J Am Mosq Control Assoc. 1987;3: 125–130. 3504901

[82] Mount GA. A critical review of ultralow-volume aerosols of insecticide applied with vehicle-mounted generators for adult mosquito control. J Am Mosq Control Assoc. 1998;14: 305–334. 9813829

[83] Barber JAS, Greer M, Coughlin J. Field tests of malathion and permethrin applied via a truck-mounted cold fogger to both open and vegetated habitats. J Am Mosq Control Assoc. 2007;23: 55–59. doi: 10.2987/8756-971X(2007)23[55:FTOMAP]2.0.CO;2 17536368

[84] Eliason DA, Campos IEG, Moore CG, Reiter MP. Apparent influence of the stage of blood meal digestion on the efficacy of ground applied ULV aerosols for the control of urban *Culex* mosquitoes. II. Laboratory evidence. J Am Mosq Control Assoc. 1990;6: 366–370. 2230762

[85] Machani MG, Ochomo E, Sang D, Bonizzoni M, Zhou G, Githeko AK, et al. Influence of blood meal and age of mosquitoes on susceptibility to pyrethroids in *Anopheles gambiae* from Western Kenya. Malar J. 2019;18: 112. doi: 10.1186/s12936-019-2746-6 30940139 PMC6444593

[86] Oliver S V., Lyons CL, Brooke BD. The effect of blood feeding on insecticide resistance intensity and adult longevity in the major malaria vector *Anopheles funestus* (Diptera: Culicidae). Sci Rep. 2022;12:3877. doi: 10.1038/s41598-022-07798-w 35264696 PMC8907345

[87] Spillings BL, Coetzee M, Koekemoer LL, Brooke BD. The effect of a single blood meal on the phenotypic expression of insecticide resistance in the major malaria vector *Anopheles funestus*. Malar J. 2008;7:226. doi: 10.1186/1475-2875-7-226 18973704 PMC2584071

[88] Benoit JB, Lopez-Martinez G, Patrick KR, Phillips ZP, Krause TB, Denlinger DL. Drinking a hot blood meal elicits a protective heat shock response in mosquitoes. Proc Natl Acad Sci U S A. 2011;108: 8026–8029. doi: 10.1073/pnas.1105195108 21518875 PMC3093486

[89] Klepeis NE, Nelson WC, Ott WR, Robinson JP, Tsang AM, Switzer P, et al. The National Human Activity Pattern Survey (NHAPS): a resource for assessing exposure to environmental pollutants. J Expo Sci Environ Epidemiol. 2001;11: 231–252. doi: 10.1038/sj.jea.7500165 11477521

[90] Wilke ABB, Damian D, Litvinova M, Byrne T, Zardini A, Poletti P, et al. Spatiotemporal distribution of vector mosquito species and areas at risk for arbovirus transmission in Maricopa County, Arizona. Acta Trop 2023; 240:106833. doi: 10.1016/j.actatropica.2023.106833 36736524

[91] Govella NJ, Johnson PCD, Killeen GF, Ferguson HM. Heritability of biting time behaviours in the major African malaria vector *Anopheles arabiensis*. Malar J. 2023;22:238. doi: 10.1186/s12936-023-04671-7 37587487 PMC10433675

[92] Yang YY, Liu Y, Teng HJ, Sauman I, Sehnal F, Lee HJ. Circadian control of permethrin-resistance in the mosquito *Aedes aegypti*. J Insect Physiol. 2010;56: 1219–1223. doi: 10.1016/j.jinsphys.2010.03.028 20361972

[93] Shetty V, Adelman ZN, Slotman MA. Effects of circadian clock disruption on gene expression and biological processes in *Aedes aegypti*. BMC Genomics. 2024;25: 170. doi: 10.1186/s12864-024-10078-8 38347446 PMC10863115

[94] Shinkawa Y, Takeda S-I, Tomioka K, Matsumoto A, Oda T, Chiba Y. Variability in circadian activity patterns within the *Culex pipiens* complex (Diptera: Culicidae). J Med Entomol. 1994;31: 49–56. doi: 10.1093/jmedent/31.1.49 8158629

[95] Fyie LR, Gardiner MM, Meuti ME. Artificial light at night alters the seasonal responses of biting mosquitoes. J Insect Physiol. 2021;129: 104194. doi: 10.1016/j.jinsphys.2021.104194 33482172

[96] Mirzabaev A, Stringer LC, Benjaminsen TA, Gonzalez P, Harris R, Jafari M, et al. Cross-chapter paper 3: Deserts, semiarid areas and desertification. In: Climate change 2022: Impacts, adaptation and vulnerability. Contribution of working group II to the sixth assessment report of the Intergovernmental Panel on Climate Change. Cambridge, UK and New York, NY, USA; 2022. doi: 10.1017/9781009325844.020

[97] Eikenberry SE, Gumel AB. Mathematical modeling of climate change and malaria transmission dynamics: a historical review. J Math Biol. 2018;77: 857–933. doi: 10.1007/s00285-018-1229-7 29691632

[98] Patil NS, Lole KS, Deobagkar DN. Adaptive larval thermotolerance and induced cross-tolerance to propoxur insecticide in mosquitoes *Anopheles stephensi* and *Aedes aegypti*. Med Vet Entomol. 1996;10: 277–82. 8887340 10.1111/j.1365-2915.1996.tb00743.x

[99] Starner K, Kuivila KM, Jennings B, Moon GE. Degradation rates of six pesticides in water from the Sacramento River, California. 1999. U.S. Geological Survey Toxic Substances Hydrology Program—Proceedings of the Technical Meeting, Charleston, South Carolina, v. 2. Contamination of hydrologic Systems and Related Ecosystems, Report 99–4018 B.

[100] Schulte PM. The effects of temperature on aerobic metabolism: Towards a mechanistic understanding of the responses of ectotherms to a changing environment. J Exp Biol. 2015;218: 1856–1866. doi: 10.1242/jeb.118851 26085663

[101] Reinhold JM, Lazzari CR, Lahondère C. Effects of the environmental temperature on *Aedes aegypti* and *Aedes albopictus* mosquitoes: A review. Insects. 2018;9:158. doi: 10.3390/insects9040158 30404142 PMC6316560

[102] Owusu HF, Chitnis N, Müller P. Insecticide susceptibility of *Anopheles* mosquitoes changes in response to variations in the larval environment. Sci Rep. 2017;7:3667. doi: 10.1038/s41598-017-03918-z 28623302 PMC5473885

[103] United States Environmental Protection Agency. Pesticides used to control adult mosquitoes. 2023 [cited 21 Dec 2023]. Available from: https://www.epa.gov/mosquitocontrol/pesticides-used-control-adult-mosquitoes

[104] Lofgren CS, Ford HR, Tonn RJ, Sujarti Jatanasen &. The effectiveness of ultra-low-volume applications of malathion at a rate of 6 US fluid ounces per acre in controlling *Aedes aegypti* in a large-scale test at Nakhon Sawan, Thailand. Bull World Health Organ. 1970;42: 15–25. 5309512 PMC2427516

[105] Reiter P, Nathan MB. Guidelines for assessing the efficacy of insecticidal space sprays for control of the dengue vector, *Aedes aegypti*. 2001. Available from: https://www.who.int/publications/i/item/who-cds-cpe-pvc-2001.1

[106] Lothrop HD, Lothrop BB, Gomsi DE, Reisen WK. Intensive early season adulticide applications decrease arbovirus transmission throughout the Coachella Valley, Riverside County, California. Vector-Borne and Zoonotic Dis. 2008;8: 475–489. doi: 10.1089/vbz.2007.0238 18494603 PMC2978539

[107] Thongsripong P, Hyman JM, Kapan DD, Bennett SN. Human-mosquito contact: A missing link in our understanding of mosquito-borne disease transmission dynamics. Annals of the Entomological Society of America.2021; 114: 397–414. doi: 10.1093/aesa/saab011 34249219 PMC8266639

[108] Stoddard ST, Morrison AC, Vazquez-Prokopec GM, Soldan VP, Kochel TJ, Kitron U, et al. The role of human movement in the transmission of vector-borne pathogens. PLoS Negl Trop Dis. 2009;3: e481. doi: 10.1371/journal.pntd.0000481 19621090 PMC2710008

[109] Monroe A, Mihayo K, Okumu F, Finda M, Moore S, Koenker H, et al. Human behaviour and residual malaria transmission in Zanzibar: Findings from in-depth interviews and direct observation of community events. Malar J. 2019;18:220. doi: 10.1186/s12936-019-2855-2 31262306 PMC6604484

[110] Agyekum TP, Arko-Mensah J, Botwe PK, Hogarh JN, Issah I, Dadzie SK, et al. Relationship between temperature and *Anopheles gambiae* sensu lato mosquitoes’ susceptibility to pyrethroids and expression of metabolic enzymes. Parasit Vectors. 2022;15:163. doi: 10.1186/s13071-022-05273-z 35527275 PMC9080126

